# Phylogeny, biogeography and ecological diversification of New Caledonian palms (Arecaceae)

**DOI:** 10.1093/aob/mcae043

**Published:** 2024-03-25

**Authors:** Victor Pérez-Calle, Sidonie Bellot, Benedikt G Kuhnhäuser, Yohan Pillon, Félix Forest, Ilia J Leitch, William J Baker

**Affiliations:** Department of Biology, Memorial University of Newfoundland, St John’s, Newfoundland A1B 3X9, Canada; Royal Botanic Gardens, Kew, Richmond TW9 3AE, UK; Royal Botanic Gardens, Kew, Richmond TW9 3AE, UK; DIADE, Univ Montpellier, CIRAD, IRD, Montpellier, France; Royal Botanic Gardens, Kew, Richmond TW9 3AE, UK; Royal Botanic Gardens, Kew, Richmond TW9 3AE, UK; Royal Botanic Gardens, Kew, Richmond TW9 3AE, UK

**Keywords:** Arecaceae, Areceae, biogeography, molecular dating, New Caledonia, phylogeny, speciation, target sequence capture, ultramafic

## Abstract

**Background and Aims:**

The geographical origin and evolutionary mechanisms underpinning the rich and distinctive New Caledonian flora remain poorly understood. This is attributable to the complex geological past of the island and to the scarcity of well-resolved species-level phylogenies. Here, we infer phylogenetic relationships and divergence times of New Caledonian palms, which comprise 40 species. We use this framework to elucidate the biogeography of New Caledonian palm lineages and to explore how extant species might have formed.

**Methods:**

A phylogenetic tree including 37 New Caledonian palm species and 77 relatives from tribe Areceae was inferred from 151 nuclear genes obtained by targeted sequencing. Fossil-calibrated divergence times were estimated and ancestral ranges inferred. Ancestral and extant ecological preferences in terms of elevation, precipitation and substrate were compared between New Caledonian sister species to explore their possible roles as drivers of speciation.

**Key Results:**

New Caledonian palms form four well-supported clades, inside which relationships are well resolved. Our results support the current classification but suggest that *Veillonia* and *Campecarpus* should be resurrected and fail to clarify whether Rhopalostylidinae is sister to or nested in Basseliniinae. New Caledonian palm lineages are derived from New Guinean and Australian ancestors, which reached the island through at least three independent dispersal events between the Eocene and Miocene. Palms then dispersed out of New Caledonia at least five times, mainly towards Pacific islands. Geographical and ecological transitions associated with speciation events differed across time and genera. Substrate transitions were more frequently associated with older events than with younger ones.

**Conclusions:**

Neighbouring areas and a mosaic of local habitats shaped the palm flora of New Caledonia, and the island played a significant role in generating palm diversity across the Pacific region. This new spatio-temporal framework will enable population-level ecological and genetic studies to unpick the mechanisms underpinning New Caledonian palm endemism.

## INTRODUCTION

New Caledonia is an archipelago located in the Southwest Pacific that is considered a biodiversity hotspot for its numerous endemic species and genera (Myers *et al.*, 2000; Pillon *et al.*, 2017), many of which are threatened by fragmented distribution, fires, mining and alien species (Veillon, 1993; Pascal *et al.*, 2008; Jaffré *et al.*, 2010). The basal stratum of the main island, Grande Terre, is a part of Gondwana that moved away from what is now Australia during the Cretaceous (Pelletier, 2007; Neall and Trewick, 2008). Geological studies suggest that New Caledonia was submerged at some point during its geological history, most likely ~60–75 Ma (Maurizot and Campbell, 2020). Regardless of the precise timing and even degree of submergence, most studies agree that the entire biodiversity of the archipelago is likely to have originated from dispersal events from other landmasses and/or from now-submerged local islands, followed by *in situ* speciation (Grandcolas *et al.*, 2008; Grandcolas, 2017; Nattier *et al.*, 2017; Malem *et al.*, 2023), as opposed to vicariance (Ladiges and Cantrill, 2007; Heads, 2023). New Caledonia is located ~1500 km east of Australia, 400 km south of Vanuatu and 2000 km north of New Zealand (Grandcolas *et al.*, 2008; Ibanez *et al.*, 2014). A phytogeographical analysis based on shared genera between landmasses has revealed strongest affinities of the New Caledonian flora with that of Australia, New Guinea and, to a lesser extent, Malesia (Morat, 1993). Biogeographical studies based on molecular phylogenetic data, usually limited in taxonomic scope (e.g. a single genus), have confirmed a likely origin of groups such as *Nothofagus* (Swenson *et al.*, 2001) or *Diospyros* (Duangjai *et al.*, 2009) in Sahul or Southeast Asia. However, few studies have investigated a large enough taxonomic group to understand the timing and direction of the dispersal of plants between New Caledonia and neighbouring landmasses (but for conifers, see Condamine *et al.*, 2017).

The factors that could have driven *in situ* speciation in New Caledonia also remain unclear. Currently, New Caledonia is best understood as a dynamic mosaic of soils and habitats that kept changing following geological and climatic events (Jaffré, 1993; Pintaud *et al.*, 2001; Grandcolas, 2017). This makes New Caledonia an ideal setting in which to explore how geology and climate can interact to promote speciation and extinction. Three main variables have been suggested to act as potential drivers of speciation for New Caledonian plant lineages (Morat, 1993; Veillon, 1993; Pintaud and Jaffré, 2001; Pintaud *et al.*, 2001; Barrabé *et al.*, 2014; Paun *et al.*, 2016). The first one is substrate heterogeneity: the soil of the main island consists mainly of a mosaic of ultramafic rocks and volcano-sedimentary substrates, whereas the soil of Loyalty Islands consists mainly of coral limestone. The second variable is precipitation heterogeneity in time and space: New Caledonia has experienced an alternation of wetter and drier episodes owing to continental drift and global climate change. Today, the eastern part of Grande Terre is more humid than the western side owing to a west–east gradient in precipitation ranging from ~800 to 4500 mm year^−1^ (Caudmont and Maitrepierre, 2007). The third variable is elevation heterogeneity in time and space, resulting from the formation of different massifs across the main island, today reaching ≤1600 m a.s.l. These three variables all create a dynamic mosaic of habitats, which might promote adaptive and/or allopatric speciation. So far, studies exploring the respective roles of different ecological factors in shaping the flora of New Caledonia have focused more on explaining distribution patterns than on explaining speciation patterns, owing to a lack of well-resolved phylogenetic hypotheses for New Caledonian clades.

Palms are a key component of the endemic flora of New Caledonia and have great potential to deliver phylogenetic insights into the evolutionary processes on the island. The family is an important component of global tropical rainforests, and multiple palm lineages have dispersed and diversified across Southeast Asia and the Pacific islands, leading to the formation of hundreds of endemic species (Dransfield *et al.*, 2008). In New Caledonia, palms are among the most dominant plant families on both ultramafic (i.e. rich in heavy metals) and non-ultramafic soils (Ibanez *et al.*, 2014). Forty native palm species are found in New Caledonia, all of which are endemic (the widespread, potentially introduced coconut, *Cocos nucifera*, is not considered here). Thirty-nine species belong to a single tribe, Areceae (subfamily Arecoideae), with the one remaining species, *Saribus jeanneneyi* (Becc.) C.D.Bacon & W.J.Baker (tribe Trachycarpeae), being the sole New Caledonian representative of subfamily Coryphoideae. The taxonomy of New Caledonian palms has been revised recently (Pintaud and Baker, 2008; Pintaud and Stauffer, 2011; Hodel *et al.*, 2021) based on molecular phylogenetics (Baker *et al.*, 2009, 2011; Baker and Couvreur, 2013), leading to the classification of the arecoid species into three subtribes and seven genera: Clinospermatinae (*Clinosperma* Becc. and *Cyphokentia* Brongn.), Basseliniinae (*Basselinia* Vieill., *Burretiokentia* Pic.Serm., *Cyphophoenix* H.Wendl. ex Hook.f. and *Cyphosperma* H.Wendl. ex Hook.f.), and Archontophoenicinae (*Chambeyronia* H.Wendl. ex Hook.f.). These genera are all endemic to New Caledonia except for *Cyphosperma*, which also occurs in Fiji and Vanuatu. Previous phylogenetic studies show that all three subtribes belong to the ‘western Pacific clade’ of tribe Areceae (Norup *et al.*, 2006; Baker *et al.*, 2011), which comprises ~210 species from 38 genera (POWO, 2023), and that the closest relatives of New Caledonian palms occur on surrounding archipelagos and landmasses, including Vanuatu, Australia, New Zealand, Fiji and the Solomon Islands (Baker *et al.*, 2009, 2011). The distribution of palms within New Caledonia has been linked to precipitation and substrate and, to a lesser extent, to elevation (Pintaud and Jaffré, 2001; Pintaud *et al.*, 2001). Drier periods during the Pleistocene and/or Tertiary are also thought to have shaped the current heterogeneity in species diversity, because two main disjunct areas of high palm diversity (in the north-east and south of Grande Terre) correspond to areas of highest rainfall that could have served as refugia during dry periods (Pintaud *et al.*, 2001). However, phylogenetic tests of these hypotheses have not been possible so far because available phylogenies are incomplete, lack resolution at the species level and are not dated, which precludes inferences of the number, direction and timing of dispersal events between New Caledonia and neighbouring regions, in addition to exploration of the role of geological and ecological factors as drivers of speciation.

The development of high-throughput, short-read sequencing now allows the inference of comprehensive species-level phylogenies for large clades because it has opened the door to large-scale sequencing of degraded DNA extracted from museum collections (Brewer *et al.*, 2019). In addition, reduced representation methods, such as target sequence capture, have recently gained popularity in the phylogenomics community because they permit the sequencing of hundreds of target DNA regions at a relatively low cost (Dodsworth *et al.*, 2019). The availability of target sequence capture kits designed for specific lineages (Nikolov *et al.*, 2019; Schneider *et al.*, 2021) or to work across many lineages (Johnson *et al.*, 2019) now renders large-scale, species-level studies affordable and informative. Applying genomic approaches to the New Caledonian system has the potential to provide new insights on the mechanisms underpinning its unique biodiversity, as illustrated by a study showing how allopatric and ecological speciation might have shaped New Caledonian *Diospyros* diversity (Paun *et al.*, 2016).

Here, we used a palm-specific target sequence capture kit (Heyduk *et al.*, 2016) to sequence the DNA of New Caledonian arecoid palms and other representatives of the western Pacific clade of Areceae from the eastern Asia–Pacific region. The unprecedented phylogenetic resolution, based on 151 nuclear loci, achieved through this approach allowed us to estimate the evolutionary time frame of New Caledonian arecoid palms, meaning that we could investigate their origins, the timing of speciation events in the archipelago and the direction of dispersal events involving New Caledonia. Finally, we inferred the soil, precipitation and elevational preferences for each species and their ancestors to explore how different ecological factors might have shaped New Caledonian palm diversity through time. This combination of phylogenomics with molecular dating and inferences of ancestral range and ecological preferences allows us to shed new light on the evolution of New Caledonian palms and the shaping of the New Caledonian flora.

## MATERIALS AND METHODS

### Taxon sampling

All 39 species of New Caledonian arecoid palms were targeted in this study, following the accepted species listed in Plants of the World Online (POWO, 2023), including new combinations made recently by Hodel *et al.* (2021). Three species belonging to the New Caledonian arecoid genera could not be included in the analyses because samples were unavailable: *Cyphosperma naboutinense* Hodel & Marcus (Fiji), *Cyphosperma voutmelense* Dowe (Vanuatu) and the recently described *Chambeyronia houailouensis* Hodel and Barrett from New Caledonia (Dowe and Cabalion, 1996; Hodel and Marcus, 2011; Hodel *et al.*, 2021). To test the monophyly of the focal genera and subtribes, we also included one to four species from all remaining genera of the western Pacific clade, at least one species of each of the seven remaining Areceae subtribes and at least one species of each of the ten genera that are still unplaced at the subtribal level in Areceae (Baker *et al.*, 2011; Baker and Dransfield, 2016). A species of tribe Euterpeae (*Euterpe edulis* Mart.) was added to serve as outgroup because this tribe has previously been shown to be closely related to Areceae (Baker *et al.*, 2009; Comer *et al.*, 2016; Matsunaga and Smith, 2021). This amounted to 113 species represented by 116 samples, which are listed in the Supplementary Data (Table S1) with their voucher and location information.

### Acquisition of genomic data

DNA extraction and DNA library preparation followed the same protocols as in the studies by Brewer *et al.* (2019) and Kuhnhäuser *et al.* (2021), respectively. Briefly, DNA was extracted from leaves dried in silica gel or sampled from herbarium specimens, following a protocol based on cetrimonium bromide (CTAB) modified from Doyle and Doyle (1987) as described by Brewer *et al.* (2019). When not already fragmented, the DNA was sonicated to yield fragments no longer than ~1000 bp using an M220 Focused-ultrasonicator™ with microTUBES AFA Fiber Pre-Slit Snap-Cap (Covaris, Woburn, MA, USA), with the following settings: peak power, 50; duty percentage factor, 20; cycles per burst, 200; power, 10; duration, 55 s; and temperature, 20 °C. Libraries were then prepared using the DNA NEBNext^®^ Ultra™ II Library Prep Kit and the NEBNext^®^ Multiplex Oligos for Illumina^®^ (Dual Index Primers Set 1) from New England BioLabs (Ipswich, MA, USA). The ‘Heyduk’ or ‘PhyloPalm’ target sequence capture RNA probe sets (Heyduk *et al.*, 2016; Loiseau *et al.*, 2019) synthesized by Arbor Biosciences myBaits^®^ (now Daicel) were then used to capture target genes by hybridizing the DNA libraries to the RNA probes for 20–24 h at 65 °C. Hybridized DNA was then washed following the manufacturer’s protocol (v.4.0; http://www.arborbiosci.com/mybaits-manual), amplified with 12–16 PCR cycles following the same protocol. Pooled libraries were then sequenced on an Illumina MiSeq with v.2 or v.3 chemistry at the Royal Botanic Gardens, Kew or on an Illumina HiSeq X (Illumina, San Diego, CA, USA) at Macrogen Inc. (Seoul, Korea), respectively. This yielded, respectively, 2 × 300 bp or 2 × 150 bp paired-end sequencing reads. The Heyduk probe set targets 176 loci, whereas the PhyloPalm set targets 971 loci, including the Heyduk ones. The kit used for each sample is indicated in the Supplementary Data (Table S1). To reduce the impact of missing data, this study uses only the 176 loci common to both probe sets, i.e. the Heyduk loci.

### Cleaning of genomic data

The reads of each sample were subjected to quality control using FastQC (Andrews, 2010). Adapters were removed using Trimmomatic (Bolger *et al.*, 2014) in ‘paired-end’ mode, allowing only one mismatch during the search for matches between the reads and the adapter sequence, with a palindrome clip threshold of 30 and a simple clip threshold of ten. Minimum adapter length was set at 2 bp, and the reverse read was retained by setting the keepBothReads option to ‘true’. Leading and trailing bases with a quality score of <20 were removed. A sliding window trimming was also performed with a window size of four bases and an average required quality score of 20. Moreover, the MINLEN option was used to discard reads with a final length of <36 bp, because short reads might lead to wrong/ambiguous assemblies. The trimmed reads were then subjected to a second quality check, also using FastQC, to make sure the trimming had worked. The median number of reads per sample was 1 300 660 reads, ranging from 1099 reads in *Cyphosperma trichospadix* (sample SBL663) to 10 770 584 reads in *Ptychococcus lepidotus* (sample SBL266).

### Gene recovery and assembly

The recovery and assembly of the sequencing reads was performed with the HybPiper pipeline (Johnson *et al.*, 2016). The pipeline used BWA (Li and Durbin, 2009) to map reads to a reference file comprising reference sequences for the target loci, and SPAdes (Bankevich *et al.*, 2012) to assemble the reads mapping to a given target region into a single contig. The reference file comprised coding sequences (CDS) of the 176 Heyduk loci (Heyduk *et al.*, 2016) obtained from *Elaeis* Jacq. (132 CDS), *Sabal* Adans. (39 CDS), *Phoenix* L. (3 CDS) and *Nypa* Steck (2 CDS), which represent three palm subfamilies, including Arecoideae. A preliminary CDS recovery was carried out on all samples with HybPiper and this reference file, setting the SPADES depth-of-coverage cut-off parameter at five and allowing for separate recovery of exons and introns. This was possible because reads do not necessarily fall exclusively in target CDS regions but can overlap off-target intronic regions, allowing us to retrieve the latter (also known as the ‘splash zone’; Johnson *et al.*, 2016). The hybpiper_stats.py script (also part of the HybPiper pipeline) was then used to calculate recovery statistics for all samples. The four genera used to make the reference file are not closely related to Areceae, which could affect the CDS recovery; therefore, a new reference file was created with the 836 exons and 754 introns recovered from the *Clinosperma bracteale* (Brongn.) Becc. Sample, which was among the samples with the highest recovery. This new *Clinosperma bracteale* reference file was then used to recover the exons and introns of the other samples, using HybPiper as before with a depth-of-coverage cut-off of five.

### Sequence alignment

For each exon and intron, the sequences from all samples were aligned with MAFFT v.7 (Katoh and Standley, 2013), using ≤1000 iterations of the E-INS-i algorithm and the option ‘*adjustdirectionaccurately*’. The resulting alignments were inspected and sorted using Geneious Prime v.2021.1.1 (https://www.geneious.com). Initially, all alignments that contained <40 % of the samples (45 taxa) were removed to reduce biases attributable to missing data (Nute *et al.*, 2019). The remaining alignments were then examined manually, and those that seemed ambiguously aligned were discarded to reduce the impact of misalignment on the phylogenetic inferences. An exception was made for two exon alignments, which were manually cleaned and kept in order that more data could be available for *Physokentia avia* H.E.Moore, for which only a few exons were recovered, and for *Physokentia thurstonii* (Becc.) Becc., for which one of these exons was the only region recovered. To ensure that gene trees would be performed on sufficiently informative sequence alignments, the exon and intron alignments of each gene were then concatenated using AMAS (Borowiec, 2016). This resulted in a total of 151 gene alignments, which were then cleaned using TAPER v.1.0.0 (Zhang *et al.*, 2021) to remove spurious sequence stretches. Default settings were used because changing these settings for a few representative alignments showed no improvement. To reduce the impact of missing data and poor alignment in gappy regions, nucleotide sites with gaps in ≥70 % of the taxa were removed using a custom script (https://github.com/sidonieB/scripts/blob/master/strip_alignment_gaps.py).

### Phylogenetic analyses

Each gene alignment was analysed separately using the maximum likelihood-based phylogenetic inference program RAxML (Stamatakis, 2014), with GTRGAMMA as the model of nucleotide substitution as recommended in the manual, generating 1000 bootstrap replicates using the rapid bootstrap algorithm and setting a seed of 12 345. All 151 gene trees were then combined in a single file, and all branches with <10 % bootstrap support were collapsed using the Newick utilities tool (Junier and Zdobnov, 2010), following recommendations from Zhang *et al.* (2018). The gene trees were processed using ASTRAL-III v.5.7.7 (Zhang *et al.*, 2018) to compute the species tree that shared the maximum number of quartets with the gene trees, where a quartet is an unrooted tree of four taxa (Reaz *et al.*, 2014). The full annotation option of ASTRAL was selected to obtain two measurements of support for the species tree topology: the local posterior probability of each branch (LPP) and the quartet support (QS), i.e. the percentage of quartets in the gene trees that agree with a branch in the species tree. The species tree was rooted on *Euterpe edulis* using the *pxrr* program of the Phyx tool (Brown *et al.*, 2017). To evaluate the impact of gene tree error on our species tree inference, we repeated the latter analysis using Weighted ASTRAL (Zhang and Mirarab, 2022), with the option astral-hybrid. This option infers a species tree based on gene trees in a similar fashion to ASTRAL-III, but it weights the branches in the gene trees based on their bootstrap support and length, thereby giving less weight to branches that might be erroneous. The resulting species tree (Supplementary Data Fig. S1) was identical to the original species tree (See Results) except for 12 poorly supported branches (LPP < 0.9). The original tree was therefore used for the molecular dating and biogeographical inferences described below. Both species trees, the gene trees and the corresponding sequence alignments are available on Zenodo (Pérez-Calle *et al.*, 2024).

### Molecular dating

The dating of a species tree based on a concatenated alignment or joint analysis of all genes would be prohibitively time consuming. The software SortaDate (Smith *et al.*, 2018) was therefore used to find the 30 genes that best represent the species tree topology. This number was selected because it resulted in a tree very similar to the one obtained with ASTRAL while still allowing the computation to be tractable. Before using SortaDate, the gene trees were rooted using a custom script (https://github.com/sidonieB/scripts/blob/master/Root_trees_general_pxrr_v4.R) and *pxrr* (Brown *et al.*, 2017). When *Euterpe edulis* was not included in a gene tree, the tree was rooted on the next most outer taxon or clade based on the topology of the species tree. The gene trees were classified by SortaDate initially by their bipartition support (proportion of bipartitions in agreement with the species tree topology); then by their tree length (sum of all branch lengths, a proxy for phylogenetic informativeness); and finally, by their root-to-tip variance in branch length (a proxy for clock-likeness). Given that the only gene that included *Physokentia thurstonii* sequences (*PPHEY856*) was not selected by SortaDate, it was added afterwards, resulting in a set of 31 genes. The best substitution model for each gene was selected using IQ-TREE (Nguyen *et al.*, 2015; Trifinopoulos *et al.*, 2016) with the ModelFinder method (Kalyaanamoorthy *et al.*, 2017; Supplementary Data Table S2). The 31 genes were then concatenated using AMAS (Borowiec, 2016) and analysed using BEAST v.1.10 (Suchard *et al.*, 2018) to obtain a dated tree. Each gene was allowed to have its own substitution model following the results of IQ-TREE. A lognormal uncorrelated relaxed clock prior was chosen to allow for substitution rate heterogeneity among branches, and the tree prior was set to ‘Speciation: Birth-Death Process’ assuming that at any point in time, each lineage can speciate or go extinct (Heath, 2011). We constrained the topology of the dated tree using the ASTRAL tree as the starting topology and disabling the subtreeslide, narrowExchange, wideExchange and wilsonBalding operators during the BEAST analysis.

The age of the Areceae crown group was calibrated with *Friedemannia messelensis* Collinson, Manch. & Wilde, a fossil from the middle Eocene oil shales stratum found in Messel, Germany and dated to be 47 Myr old (Collinson *et al.*, 2012). This was supported by the morphology-based placement of this fossil in crown Areceae recovered in a previous study (Matsunaga and Smith, 2021). Given that fossils can provide only a minimum age for the lineage to which they are assigned, the age of the Areceae crown group was allowed to be older than the fossil by setting the age prior to a gamma distribution with shape = 1.0, scale = 16.0 and offset = 47.0 Ma, meaning that its median would be 58.1 Ma but its 99 % upper quantile fell conservatively at ~121 Ma, corresponding to the age of the oldest known palm fossils (*Spinizonocolpites* pollen from the Albian or possibly the Aptian–late Barremian of Patagonia, Argentina, most probably representing the stem group of palms; Martínez *et al.*, 2016). The relaxed clock prior (‘ucld.mean’) was set to a gamma distribution with shape = 0.001 and scale = 1000, following the recommendation of Drummond and Bouckaert (2015). The Bayesian inference was run for 200 million generations, saving a tree every 20 000 generations. Three independent runs were performed. Checks using Tracer v.1.7.1 (Rambaut *et al.*, 2018) indicated that the three runs converged towards the same parameter estimates, and all effective sample size (ESS) values of the individual runs were >200, except for treeModel.rootHeight and coefficientOfVariation (ESS > 100). The 1500 (15 %) first trees corresponding to the burn-in phase of the analysis were removed from each run, and the remaining trees from the three runs were combined using LogCombiner v.1.10.4. The ESS of all parameters estimated from the combined posterior tree distributions were >200. A maximum clade credibility tree with median node heights was then built from the combined posterior trees using TreeAnnotator v.1.10.4 and visualized and edited with FigTree v.1.4.4 (http://tree.bio.ed.ac.uk/software/figtree/).

### Ancestral range inferences

The inference of ancestral species ranges was made using the BioGeoBEARS R package v.1.1.2 (Matzke, 2013). To simplify the number of areas and to keep the focus on the western Pacific clade, all taxa outside this clade + *Hydriastele* H.Wendl. & Drude were removed from the dated tree using the *drop.tip()* function of the R package *ape* v.5.5 (Paradis and Schliep, 2019). These packages, and other R packages cited below, were used in R v.4.0.3 (R Core Team, 2020) through RStudio Desktop v.1.3.1093 (RStudio Team, 2020). The DEC (dispersion–extinction–cladogenesis), DIVALIKE (dispersal–vicariance analysis) and BAYAREALIKE (Bayesian analysis) biogeographical models implemented in BioGeoBEARS were compared, with and without using the founder-event speciation parameter (+J). Critique of the parameter +J (Ree and Sanmartín, 2018) has been soundly rebutted (Matzke, 2022). Therefore, we included it in some of our models because many of the taxa studied here are island taxa, a case where the +J model might be particularly suitable (Ree and Sanmartín, 2018). Eleven different areas were defined: Australia (A), Borneo (B), Caroline Islands (K), Fiji (+ Samoa; F), New Caledonia (C), New Guinea (including Bismarck archipelago; N), the Philippines (including Ryukyu Island; P), Solomon Islands (O), Southern Zealandia (including Lord Howe Island, New Zealand, Kermadec Islands; S), Vanuatu (V) and Wallacea (W) (see Results; Supplementary Data Table S1). Southern Zealandia was not divided further in order to keep the analysis computationally tractable and because exploring dispersals between its components was beyond the scope of this New Caledonia-focused study. Ancestral ranges were allowed to span a maximum of three areas at the same time.

Two sets of analyses were carried out: (1) an unconstrained analysis using only data on the current presence/absence of each species in each area; and (2) a constrained analysis, in which dispersal between areas was set as a function of the current distance separating them (Van Dam and Matzke, 2016). For the analyses using distance between areas, the distance between the nearest coasts of two areas was estimated using the ‘Measure distance’ option of Google Maps (https://www.google.com/maps/). For Australia, only the coasts of Northern Territory, Queensland and New South Wales were used (reflecting the distribution of our Australian species). For areas with a high number of islands, only the largest island was used to measure the distances (e.g. Grande Terre for New Caledonia). We did not perform time-stratified analyses owing to the uncertainty remaining regarding the geological past of the region. The constrained and unconstrained analyses were run for all three biogeographical models, with or without implementing the +J parameter, resulting in a total of 12 models that were compared based on their corrected Akaike information criterion (AICc).

### Retrieval of spatial occurrence data

Global spatial occurrence data for all New Caledonian taxa included in this study (Supplementary Data Table S1) were sourced from a previously published dataset of palm occurrences including clean data retrieved from the Global Biodiversity Information Facility (GBIF; derived dataset GBIF.org, https://doi.org/10.15468/dd.at82kf; Bellot *et al.*, 2022) and from the specimen databases of the Royal Botanic Gardens, Kew and of the Naturalis Biodiversity Center, Leiden (The Netherlands) (Bellot *et al.*, 2022). Coordinates falling into marine areas, biodiversity institutions, cities, or province or country centroids were identified and removed using the R package *CoordinateCleaner* v.1.0-7 (Zizka *et al.*, 2019). Coordinates corresponding to longitude or latitude zero, with an uncertainty of >100 km, inconsistent with country assignment, falling outside the native distribution range of the species (POWO, 2018) or recorded before 1945 (when the precision of geo-localization devices was poor) were also removed, as well were duplicated occurrences (see details in the study by Bellot *et al.*, 2022). Additional well-curated occurrence data derived from herbarium specimens and field observations and used to perform IUCN Red List assessments of threatened New Caledonian flora by the local group of botanical expertise ‘RLA Flore NC’ (e.g. Amice *et al.*, 2017*a*, *b*, *c*, *d*, 2020*a*, *b*, *c*) were obtained from the New Caledonia Red List working group (J. Maura, pers. com. 14 March 2017; Meyer *et al.*, 2022) after being aggregated to a 500 m^2^ resolution.

### Ecological analyses

To explore which ecological factors among annual precipitation, elevation and geological substrate might have played a role in driving speciation of palms in New Caledonia, the current distribution and ecological preferences of each New Caledonian species were analysed in a phylogenetic context. Annual precipitation and elevation for each species occurrence point were extracted from 1-km-resolution layers from WorldClim (Fick and Hijmans, 2017) and from the Shuttle Radar Topography Mission (Farr *et al.*, 2007), respectively, using the spatial occurrence data mentioned in the previous subsection. The function *extract()* of the R package *raster* v.3.4.5 (Hijmans, 2022) was used to extract the values from the raster layers. Substrate preferences and current distribution were obtained for each New Caledonian species from the studies by Hodel and Pintaud (1998), Pintaud and Jaffré (2001) and Pintaud and Stauffer (2011). Precipitation and elevation values for each occurrence can be found in the Supplementary Data (Table S3).

To study ecological preferences and geographical distribution in a phylogenetic context, the species tree obtained from the ASTRAL analysis was used to identify pairs of sister species to compare. Precipitation and elevation ranges between sister taxa were then compared statistically with unpaired Student’s *t*-test. Substrate preference was treated as a discrete character with three states: ‘ultramafic’, ‘schistose’ and ‘limestone’, with species allowed to present multiple states. The ‘schistose’ state encompasses any non-ultramafic volcano-sedimentary soil present on Grande Terre. A situation where one species grew in two geological substrates and its sister species grew in only one substrate was treated as a difference in extant substrate preferences. We also recorded whether the sister species were ‘sympatric’, when the distribution range of one of the sister species was overlapped by ≥50 % by the distribution range of the other; ‘allopatric’, when the distribution ranges of both sister species did not overlap; or ‘parapatric’, when the distribution ranges of the sister species overlapped in <50 % of their respective ranges. Species with a parapatric distribution were considered to have a difference in their distribution range. In a second iteration, the ecological and geographical ranges of the closest relative to each species pair were also compared with the ecological and geographical range represented by the pair. Finally, to estimate ancestral ecological preferences, the species tree obtained from the ASTRAL analysis was simplified to have only the New Caledonian species, using the *drop.tip()* function of *ape* v.5.5 (Paradis and Schliep, 2019). The function *contMap()* of the *phytools* v.0.7.70 package (Revell, 2012) was then used to estimate ancestral precipitation and elevation preferences and to plot ancestral and current preferences on the tree. Ancestral substrate preferences were inferred on the complete western Pacific clade species tree through a Markov chain Monte Carlo Bayesian search using RevBayes v.1.2.1, following a model of equal rates of transition between states as specified in the software tutorial (https://revbayes.github.io/tutorials/morph_ase/ase.html). The prior distribution for the rate of transition between states was set to an exponential distribution with a mean of ten, following the tutorial’s recommendation in the absence of prior knowledge. The substrate of species outside New Caledonia was encoded as missing. Two runs of 25 000 generations were performed. Their posterior distributions of ancestral states were combined after removing the first 25 % (burn-in fraction) and checking for convergence in Tracer (Rambaut *et al.*, 2018), and the posterior probabilities of the different states were visualized on the phylogeny using RevGadgets (Tribble *et al.*, 2022) as explained in the tutorial.

## RESULTS

### Phylogenetic relationships among New Caledonian arecoid palms and their close relatives

**Fig. 1.**
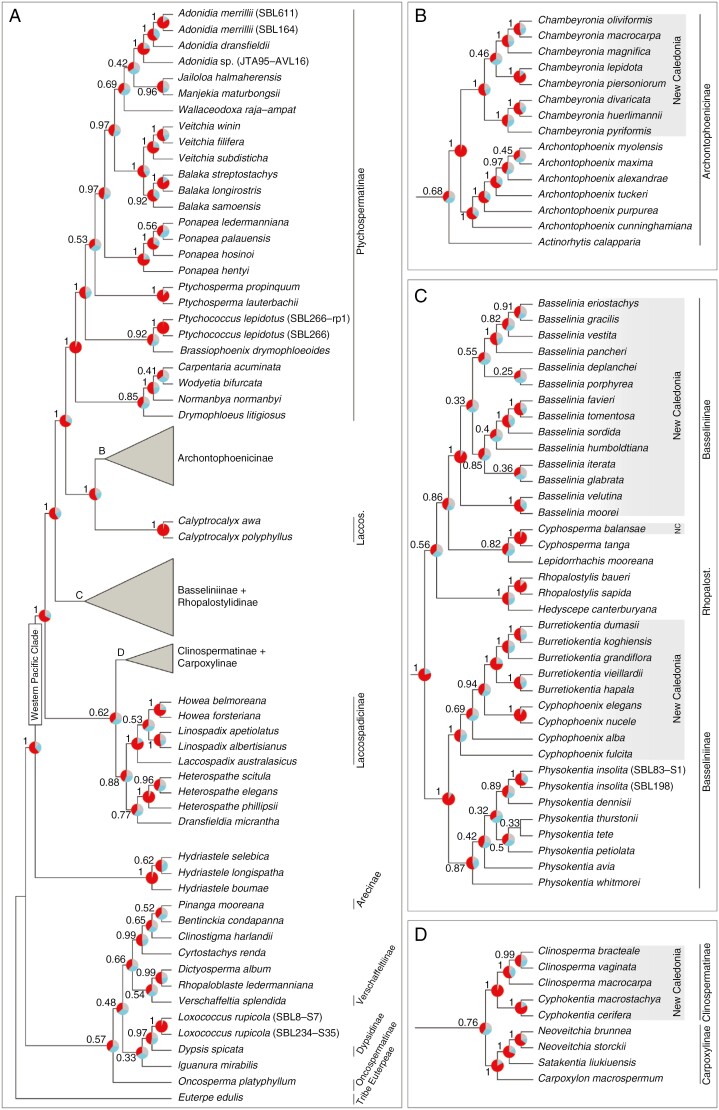
Phylogenetic relationships among New Caledonian arecoid palms and closely related genera obtained by multispecies coalescent summary analysis of 151 genes. All New Caledonian arecoid species were included in the tree except for *Clinosperma lanuginosa* (sequencing failure) and *Chambeyronia houailouensis* (not described at the time of the study). Numbers represent the local posterior probability of each clade, while pie charts show quartet support of the displayed topology (red), of the first alternative topology (blue) and of the second alternative (grey). New Caledonian lineages are highlighted in grey, with the current taxonomic classification added on the right. *Cyphophoenix alba* and *Cyphophoenix fulcita* were previously *Veillonia alba* and *Campecarpus fulcitus*, respectively. Genera without a subtribe name are unplaced according to Baker and Dransfield (2016). Abbreviations: Laccos., Laccospadicinae; NC, New Caledonia; Rhopalost., Rhopalostylidinae.

Targeted sequencing generated enough data for all sampled taxa to be included in the phylogenetic tree, with the exception of *Clinosperma lanuginosa* (Supplementary Data Table S1). Species relationships obtained from the multispecies coalescent summary analysis of 151 genetic regions were, for the most part, highly supported inside the western Pacific clade (64 of 95 nodes with LPPs ≥ 0.90), especially relationships between subtribes and genera (Fig. 1). The western Pacific clade was resolved as monophyletic, with *Hydriastele* as its sister group (LPP = 1; Fig. 1). In this clade, four, possibly five (*Cyphosperma balansae*), separate New Caledonian clades were resolved, each most closely related to a clade from Australia, Southern Zealandia (Lord Howe Island + New Zealand), Fiji or the Solomon Islands. Ptychospermatinae and Archontophoenicinae were each monophyletic (LPPs = 1 and 0.68, respectively), and they formed a maximally supported clade with *Calyptrocalyx* (Laccospadicinae), which itself grouped with Archontophoenicinae with maximal support (Fig. 1A). A close relationship between Basseliniinae and Rhopalostylidinae was maximally supported, as was their grouping closer to the clade Ptychospermatinae + Archontophoenicinae + *Calyptrocalyx* than to the remainder of the western Pacific clade (Fig. 1A). The remainder of Laccospadicinae (i.e. *Laccospadix*, *Linospadix* and *Howea*) were monophyletic (LPP = 1) and grouped with *Heterospathe* and *Dransfieldia* (LPP = 0.88), which are currently unplaced in any subtribe but are resolved here as sister genera with low support (LPP = 0.77; Fig. 1A). The clade formed by these five genera was resolved as sister to Clinospermatinae and Carpoxylinae, albeit with low support (LPP = 0.62; Fig. 1A).

Most relationships among New Caledonian species and between them and their closest relatives from outside New Caledonia were moderately (LPP ≥ 0.80) or highly (LPP ≥ 0.90) supported (Fig. 1B–D). In Archontophoenicinae, *Archontophoenix* was maximally supported as sister to *Chambeyronia*, which was monophyletic with maximal support, and all but one relationship between *Chambeyronia* species had maximal support (Fig. 1B). In this genus, *Chambeyronia divaricata* and *Chambeyronia huerlimanii* were sister and, together, sister to *Chambeyronia pyriformis*; *Chambeyronia macrocarpa* and *Chambeyronia oliviformis* were sister and, together, sister to *Chambeyronia magnifica*; and *Chambeyronia lepidota* and *Chambeyronia piersoniorum* were sister, but their relationship to the other species had low support (LPP = 0.46; Fig. 1B). In Basseliniinae and Rhopalostylidinae, all genera were resolved as monophyletic with moderate (*Physokentia*: LPP = 0.87) or maximal support, with the exception of *Cyphophoenix*, which formed a grade around *Burretiokentia* with high support (LPP > 0.94; Fig. 1C). These two genera were sister to *Physokentia* with maximal support, while *Cyphosperma* and *Lepidorrhachis* grouped together with moderate support (LPP = 0.82) and as sister to *Basselinia*, also with moderate support (LPP = 0.86). Rhopalostylidinae were monophyletic (LPP = 1) but nested inside Basseliniinae with low support (LPP = 0.56), because they were sister to *Basselinia* + *Cyphosperma* + *Lepidorrhachis* (Fig. 1C). In Basseliniinae, intrageneric relationships were generally well resolved, with the exception of *Physokentia*, where most relationships were poorly supported (LPP = 0.32–0.89; Fig. 1C). In *Burretiokentia*, all relationships had maximal support, with *Burretiokentia dumasii* and *Burretiokentia koghiensis* being sisters and themselves sister to *Burretiokentia grandiflora*, and with *Burretiokentia vieillardii* and *Burretiokentia hapala* being sisters and themselves sister to the rest of the genus. *Cyphophoenix elegans* and *Cyphophoenix nucele* were sisters with maximal support and themselves grouping with *Burretiokentia* (LPP = 0.94), while *Cyphophoenix alba* and *Cyphophoenix fulcita* branched successively outside of this group (LPP = 0.69; Fig. 1C). Inside *Basselinia*, *Basselinia gracilis* and *Basselinia eriostachys* were sisters (LPP = 0.91), themselves sister to *Basselinia pancheri* and *Basselinia vestita* (LPP = 1), with the latter possibly closer to them (LPP = 0.82); *Basselinia moorei* and *Basselinia velutina* were maximally supported as sisters, and *Basselinia tomentosa* and *Basselinia favieri* were sisters and themselves sister to *Basselinia sordida* with maximal support, while the position of the other species remained unclear (Fig. 1C). Finally, Clinospermatinae and Carpoxylinae were each maximally supported as monophyletic, and their grouping was weakly supported (LPP = 0.76; Fig. 1D). Inside Clinospermatinae, *Cyphokentia* and *Clinosperma* were each maximally supported as monophyletic, and relationships within these genera were all highly supported, with *Clinosperma vaginata* and *Clinosperma bracteale* grouping closer to each other than to *Clinosperma macrocarpa* (LPPs 0.99–1; Fig. 1D).

### Biogeographical history of New Caledonian arecoid palms

**Fig. 2.**
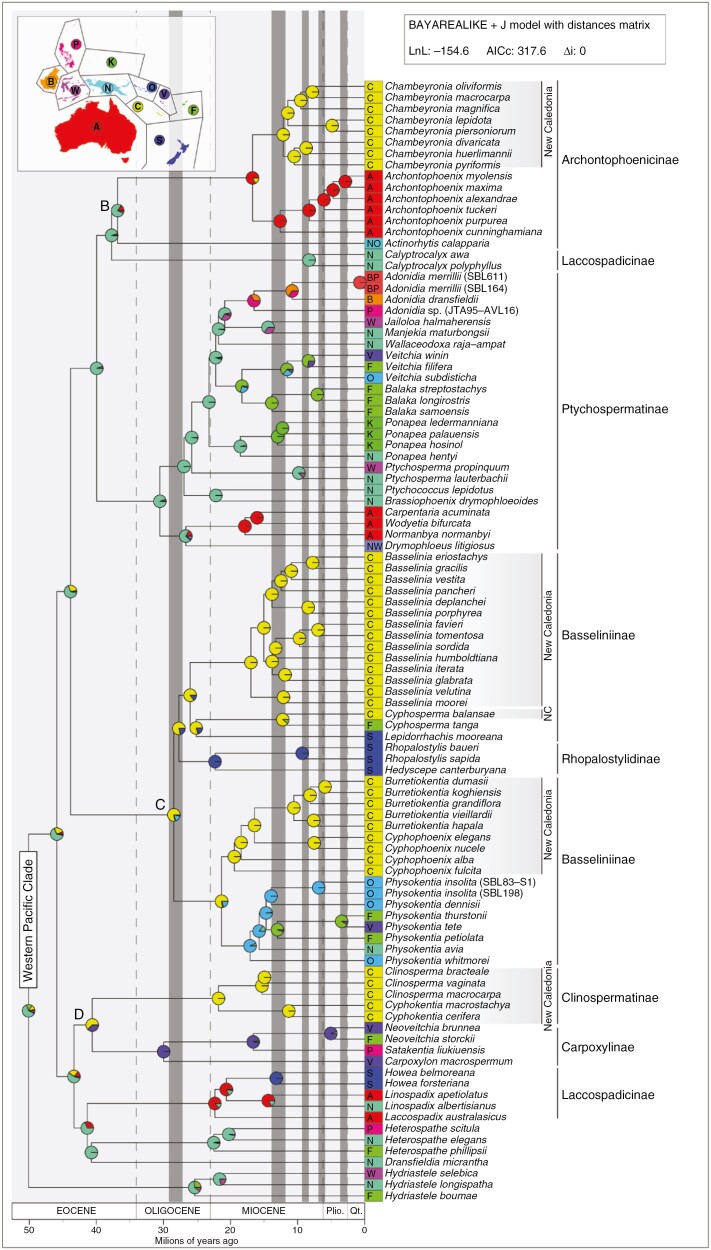
Divergence times and ancestral ranges of New Caledonian arecoids and closely related genera. Ancestral ranges were inferred under a BAYAREALIKE+J model including an area distance matrix. Pie charts represent the probability of each state, with areas colour coded as shown on the top left map. Tip squares indicate the current distribution of each taxon, with area colour and letter coded following the top left map and as described in the Materials and Methods and the Supplementary Data (Table S1). New Caledonian lineages are highlighted in grey, and the current taxonomic classification is specified on the right. Genera without a subtribe name are unplaced according to Baker and Dransfield (2016). Grey vertical bars indicate dry periods in the Southwest Pacific following Chamley (1986). The letters B, C and D indicate the clades shown in panels B, C and D of Fig. 1.

The two best biogeographical models (i.e. two models with the smallest AICc) were BAYAREALIKE+J (AICc: 317.6) and DEC+J (AICc: 323.5), both including an area distance matrix. The first and second models had a difference of AICc (∆i) 4 ≤ ∆i ≤ 7, therefore showing ‘considerably less support’ for the second model according to Burnham and Anderson (2004). Other models had ∆i ≥ 8.8 compared with the first model (Supplementary Data Table S4), therefore ‘essentially no support’ (Burnham and Anderson, 2004). Results provided by the two best models were congruent for all but six nodes (Supplementary Data Fig. S2). Five of these nodes involved undersampled genera (i.e. *Hydriastele* and *Heterospathe*) or were located towards the root, where uncertainty was higher. Given that our sampling did not allow a robust inference of the ancestral ranges outside the western Pacific clade, we only describe the historical biogeography of the New Caledonian arecoid palms and of their closest relatives. Our analysis suggests that there have been at least three colonization events for New Caledonia since the Late Eocene and that New Caledonia acted as a source from where lineages dispersed to other areas at least five times. The western Pacific clade was inferred to have diverged from the *Hydriastele* lineage ~50 Ma (95 % highest posterior density interval: 43–70 Ma; Supplementary Data Fig. S3) and its most recent common ancestor to have occurred in New Guinea (Fig. 2). The first dispersal to New Caledonia appears to have originated from New Guinea and to have involved the stem lineage of Basseliniinae + Rhopalostylidinae, between ~28 (22–40) and ~44 (35–60) Ma. A second dispersal from New Guinea to New Caledonia might have taken place in the Late Eocene before the stem lineage of Clinospermatinae and Carpoxylinae was formed, between ~40 (32–57) and ~43 (35–60) Ma (but see below about the possibility for a later dispersal from Vanuatu instead). Finally, our analysis suggests that the last colonization of New Caledonia by arecoid palms occurred during the Middle Miocene from Australia, after the divergence of the *Chambeyronia* lineage from the *Archontophoenix* lineage, between ~12 (8–17) and 17 (12–25) Ma. After their initial colonization of New Caledonia, Basseliniinae + Rhopalostylidinae appear to have dispersed at least four times out of New Caledonia. The ancestor of Rhopalostylidinae dispersed to Southern Zealandia between ~28 (21–39) and ~22 (14–33) Ma, and *Lepidorrhachis* also dispersed there at most ~25 (19–36) Ma. Then, the ancestor of *Physokentia* dispersed to the Solomon Islands between ~17 (12–24) and ~21 (16–30) Ma, and *Cyphosperma* dispersed to Fiji at most ~12 (6–20) Ma. A fifth dispersal out of New Caledonia, this time to Vanuatu, involved the ancestors of Carpoxylinae between ~30 (21–44) and ~40 (32–57) Ma, but this remains to be confirmed given that another scenario not involving a dispersal out of New Caledonia also received a relatively high, albeit lower, probability (Fig. 2). In addition to these dispersals in and out of New Caledonia, our results suggest that the history of the western Pacific clade was punctuated by at least three dispersals from New Guinea to Australia (Laccospadicinae, *Archontophoenix* and *Normanbya* + *Wodyetia* + *Carpentaria* lineages), three dispersals from New Guinea to Wallacea (*Drymophloeus*, *Jailoloa* and *Ptychosperma propinquum* lineages), one dispersal from Australia to Lord Howe island (genus *Howea*) and two dispersals from Fiji to Vanuatu (within the genera *Physokentia* and *Veitchia*).

The speciation of the extant New Caledonian species appears to have started during the Miocene (Fig. 2), and subsequent speciation events within New Caledonian clades coincide to some extent with dry periods. For example, the divergence of *Burretiokentia* from *Cyphophoenix* ~19 (14–27) Ma and of *Burretiokentia* species from each other between 11 (7–16) and 6 (3–10) Ma, respectively, overlapped with the Mid-Miocene and the Miocene–Pliocene dry periods (Fig. 2). *Clinosperma* and *Cyphokentia* species formed ~15 (8–24) and 11 (6–18) Ma, respectively, and *Chambeyronia* started to diversify ~12 (8–17) Ma, all three events overlapping with the Mid-Miocene dry period. *Basselinia* started to diversify ~17 (13–24) Ma, with eight speciation events occurring around the Mid-Miocene dry period, and the most recent species of this genus appeared ~7 (4–11) Ma, coinciding with the Miocene–Pliocene dry period (Fig. 2). Only six *Basselinia* species have their distribution range completely outside of the wettest areas in New Caledonia (Supplementary Data Fig. S4), and five of those species diverged from their sister lineage around the Miocene–Pliocene dry period, *Basselinia vestita* being the only exception (Fig. 2).

### Transitions in geographical range and ecological preferences

**Fig. 3.**
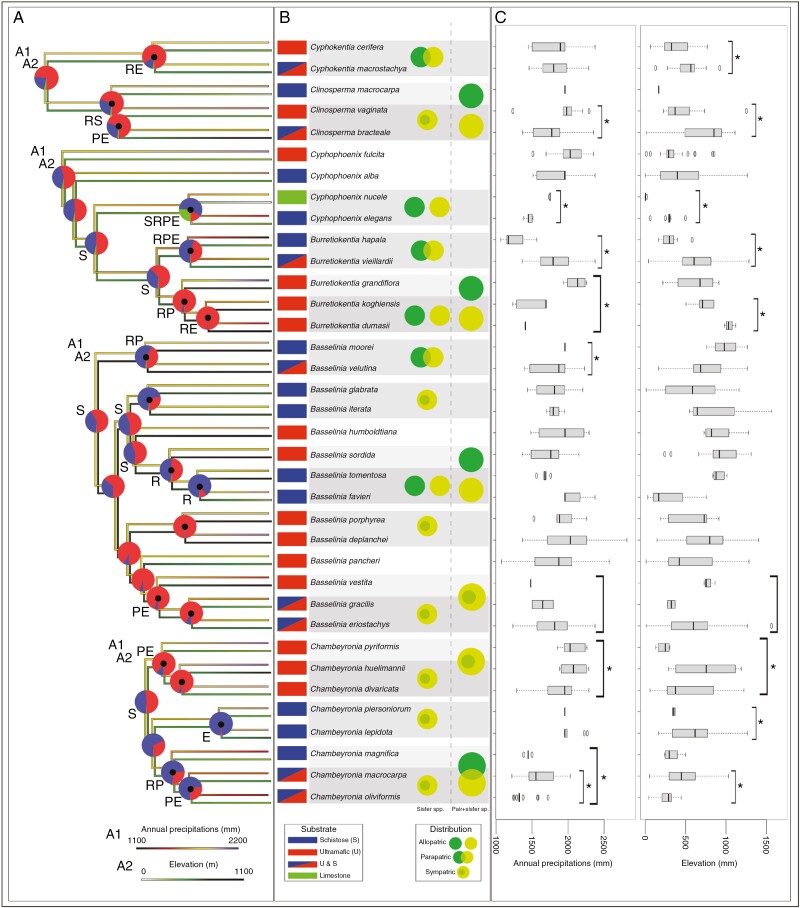
Current and ancestral ecological preferences and current distribution overlap for New Caledonian arecoid species. (A) Ancestral precipitation (top trees; A1), elevation (bottom trees; A2) and substrate (pie charts) preferences. Letters indicate transitions in precipitation (P) and elevation (E) preference based on significant differences shown in C, and transitions in range (R) and substrate (S) preference based on differences shown in B. Black dots identify speciation events for which the presence of transitions in elevation, precipitation and/or distribution range could be investigated, i.e. most recent common ancestors of sister species or of species pairs and their closest relative. (B) Current substrate preference (rectangles) and overlaps in distribution (circles). (C) Annual precipitation (in millimetres) and elevation (in metres) preferences of each species. Asterisks indicate a statistically significant difference according to Student’s *t*-test (*P*-value < 0.05). Horizontal highlighting distinguishes species pairs for easier visualization. The ‘schistose’ state encompasses any non-ultramafic volcano-sedimentary soil present on Grande Terre.

As a first step towards evaluating the role that ecological variables might have played in the speciation of New Caledonian arecoid palms, we surveyed 19 speciation events (indicated by a black dot in Fig. 3) for the presence of transitions in geographical range, substrate, precipitation and/or elevation preference. This was based on inferences of ancestral substrate and climatic preferences and on comparisons between extant ecological preferences between sister species and between species pairs and their closest relative, when the latter was a single species. To remain conservative, only past transitions in elevation or precipitation preferences that were associated with statistically significant differences in current preferences were considered; the exact timing and direction of the transitions were not inferred; and only the minimum number of transitions needed to explain the data was considered. Among the 19 speciation events considered, ten involved a transition in range. Among these, two did not involve any statistically significant ecological transition, three involved a transition in precipitation only, two in elevation only, one in substrate only, one in precipitation and elevation, and one in all three factors (Fig. 3). Among the nine speciation events that did not involve range transitions, four did not involve any statistically significant ecological transition, one involved a transition in elevation only, and four involved a transition in precipitation and elevation preference (Fig. 3). In addition to these, 13 older speciation events could be assessed for substrate transitions only (nodes without a black dot in Fig. 3), among which six were associated with such transitions. These results indicate that no one factor was more closely associated with speciation events than any others. However, some tendencies could be observed. Substrate transitions tended to be associated primarily with older speciation events, across the different genera. All but one of the speciation events in *Chambeyronia* were associated with transitions in elevation and/or precipitation preference in a context of sympatry, and all but one speciation event leading to extant species of *Cyphokentia*, *Clinosperma*, *Burretiokentia* and *Cyphophoenix* involved a range transition together with diverse ecological transitions (Fig. 3). Of the four speciation events leading to extant species that could not be associated with any ecological or geographical transitions, three were found in *Basselinia*, and transitions in elevation and precipitation were recovered only once in this genus, the largest of New Caledonian palms (Fig. 3).

## DISCUSSION

### On the relationships of New Caledonian palms

Our study provides the first comprehensive phylogenetic hypothesis for New Caledonian palms based on high-throughput sequencing data from the nuclear genome. Although some recent studies have focused on Archontophoenicinae (Domenech *et al.*, 2014; Cumberledge *et al.*, 2020; Hodel *et al.*, 2021), no study included a representative sampling of Basseliniinae, and the last comprehensive phylogenetic study of Clinospermatinae by Baker *et al.* (2011) was based on only two nuclear genes and yielded limited resolution in this group. Our results have already contributed to the taxonomic changes made by Hodel *et al.* (2021), who synonymized *Actinokentia* and *Kentiopsis* into an expanded *Chambeyronia*. Moreover, we confirm here the polyphyly of Laccospadicinae suggested by previous studies (Norup *et al.*, 2006; Baker *et al.*, 2009, 2011; Yao *et al*., 2023), with *Calyptrocalyx* being here strongly supported as sister to Archontophoenicinae (Fig. 1A). Our phylogenetic analyses also demonstrate the paraphyly of *Cyphophoenix*, indicating that the sinking of *Campecarpus fulcitus* and *Veillonia alba* in *Cyphophoenix* made by Pintaud and Baker (2008) was premature. This taxonomic change was made based on morphological cladistic analyses and limited molecular phylogenetic evidence (Pintaud and Baker, 2008), which is now superseded by our more comprehensive genomic dataset. Both *Veillonia* and *Campecarpus* can reasonably be treated as accepted genera once again. Finally, our results also imply that Rhopalostylidinae might be nested inside Basseliniinae, as suggested by (Baker *et al.*, 2009), but support for this relationship was very low (LPP = 0.56) and requires further research (Fig. 1C). Despite using a large amount of information (151 genes), some relationships among *Basselinia* species also remain unclear. In most cases, this is probably attributable to incomplete lineage sorting in the context of a relatively rapid radiation. This interpretation is supported by the short branch lengths associated with these nodes (Fig. 2) and by the fact that the two alternative topologies at the pertinent nodes are found with similar frequencies among the gene trees, which is expected when incomplete lineage sorting is the only source of conflict (Sayyari and Mirarab, 2016). However, in two deeper nodes of the genus (those with LPP = 0.33 and 0.55 in Fig. 1C), one of the alternative topologies was recovered much more than the other, pointing towards sources of conflict other than incomplete lineage sorting, such as hybridization (Degnan and Rosenberg, 2009).

### New Caledonia: recurrent sink and source of palm lineages

Our study shows that New Caledonia acted as a sink for arecoid palm lineages at least three times, with lineages dispersing twice from New Guinea to New Caledonia at different times during the Eocene/Oligocene and once from New Guinea to Australia to New Caledonia during the Miocene (Fig. 2). To this should be added two events of colonization of New Caledonia by the coryphoid palm *Saribus jeanneneyi* and by the coconut, the origins and timings of which remain to be inferred. New Caledonia also acted multiple times as a source of palm lineages for other regions, notably for *Physokentia* and *Lepidorrhachis*, and possibly also for *Cyphosperma*. However, the latter remains to be confirmed by an analysis including the three species from this genus for which we could not generate data owing to lack of material: one from Vanuatu (*Cyphosperma voutmelense*) and the other two from Fiji (*Cyphosperma naboutinense* and *Cyphosperma trichospadix*). Our sampling for western Pacific clade genera outside New Caledonia was representative of the distribution range of these genera, with missing regions attributable to undersampling occurring in only three genera (Maluku: 1 of 28 species in *Calyptrocalyx;* Australia and/or Solomon Islands: 3 of 29 species in *Ptychosperma*; Solomon islands: 9 of 39 species in *Heterospathe*). Future studies including all species from these genera will enable a more complete understanding of their biogeographical history but are unlikely to alter the role of New Caledonia as a sink and source of palm diversity significantly. Our results are in line with previous evidence that New Caledonia served as a source of lineages to neighbouring Melanesian islands for multiple angiosperm genera, notably *Kermadecia s.l.* (Mast *et al.*, 2008), *Geissois* (Pillon, 2011), *Plerandra* (Plunkett and Lowry, 2012) and *Oxera* (Barrabe *et al.*, 2015), and for conifers (Condamine *et al.*, 2017). Dispersal from New Caledonia to Southern Zealandia is less commonly inferred, but dispersal to New Zealand was suggested for *Litsea* (Munzinger *et al.*, 2023). In contrast, species-rich palm genera such as *Calamus* and *Licuala*, which are widespread in Southeast Asia and present in Vanuatu or Fiji, are not currently found in New Caledonia. This situation has been observed in other plant families, such as Gesneriaceae, where tribe Coronanthereae is well represented in New Caledonia (Woo *et al.*, 2011) whereas the genus *Cyrtandra*, widespread in Southeast Asia and the Pacific, is represented in New Caledonia by a single species restricted to the Loyalty Islands (Johnson *et al.*, 2017). New Caledonia thus appears to have served as a springboard into the Pacific for some Asian lineages but to have resisted colonization by other lineages that dispersed from Asia to the Pacific via stepping stones formed by the Melanesian island chain.

### Divergence time estimates are compatible with the geological history of Melanesia

New Caledonian palms are older than previously inferred by Baker and Couvreur (2013). This could be attributable to the use of a different fossil (*Friedemannia messelensis*) placed with high support in the crown of Areceae by Matsunaga and Smith (2021), our denser sampling (Bromham, 2019) and/or the use of different prior distributions in the dating analysis. The stem age of the oldest New Caledonian palm clade, Clinospermatinae, matches with the re-emergence of Grande Terre ~37 Ma or earlier (Fig. 2) (Pelletier, 2007; Maurizot and Campbell, 2020), in line with what was found for other plant groups, such as *Pycnandra* in Sapotaceae (Pillon, 2012). The most recent common ancestor of Basseliniinae was inferred to have occurred in New Caledonia at the latest 28 (22–40) Ma (Fig. 2). Considering that New Caledonian palms are mostly forest dwellers (Hodel and Pintaud, 1998), it can be hypothesized that forests on Grande Terre are at least that old. Most of New Guinea emerged ~10 Ma, with the mountains rising within the last 5 Myr (Hall, 2009, 2012, 2017). However, we found the New Guinean ancestors of the New Caledonian lineages to be older than this, in line with new tectonic reconstructions indicating that parts of New Guinea were above water long before the Miocene (Gold *et al.*, 2020). All dispersals to Fiji and the Solomon Islands were found to have happened in the late Oligocene and in the Miocene, in agreement with studies showing that these islands started to emerge between the Late Eocene and the Oligocene (Neall and Trewick, 2008; Holl, 2013; Haase *et al.*, 2020). We found that the dispersal of *Ponapea* from New Guinea to the Caroline Islands could have happened at the latest ~13 (8–20) Ma, which is compatible with the formation of the Main Chuuk Lava Series responsible for their emergence 12 Ma (Rehman *et al.*, 2013). Our finding that Carpoxylinae dispersed from New Caledonia to Vanuatu at the latest ~30 (21–44) Ma is somewhat compatible with the complex geological history of the area, because the old islands arc across the Vitiaz Trench (now submerged) appear to have formed after the Late Oligocene, although the current islands of the New Hebrides arc originated ~10 Ma onwards (Haase *et al.*, 2020).

### Potential drivers of speciation in New Caledonian palms

The presence of associations between speciation events, past large-scale environmental changes (e.g. drier periods) and transitions in lineage range or habitat (e.g. precipitation, elevation and/or substrate preferences) should not be mistaken for definitive proof that these factors drove diversification. However, describing such associations can help to infer which factors might have played a more prominent role than others in driving past speciation events or at least in contributing to species differentiation by creating barriers against gene flow. Based on median ages, speciation events in New Caledonian palms were found to be concentrated during the Middle to Late Miocene, specifically around the periods of drier tropical climate known to have occurred in the Southwest Pacific at that time (Chamley, 1986; Chevillotte *et al.*, 2006). This supports the hypothesis of Pintaud *et al.* (2001) that areas of highest rainfall could have served as refugia and promoted speciation during Pleistocene and/or Tertiary dry periods. However, this apparent coincidence between speciation events and dry periods should be considered with caution owing to the relatively large confidence intervals around divergence time estimates (Supplementary Data Fig. S3). The timing of speciation events and the current distribution of sister species (see Figs 2 and 3; Supplementary Data Fig. S4) suggest that, if speciation was caused by isolation during dry periods, it would have taken place in the Miocene and would have been followed by dispersal outside refugia.

Geographical isolation could have played a role in the diversification of all New Caledonian palm genera but was apparently more frequent in *Burretiokentia* and less so in *Basselinia* and *Chambeyronia*, despite their higher number of species (Fig. 3). Transitions in range, in elevation and in precipitation preferences were associated with almost the same number of speciation events (ten, nine and nine, respectively), illustrating the absence of dominance of one of these factors over the others (Fig. 3). Transitions in substrate preference were rare (only two cases) among the speciation events also surveyed for transitions in range, elevation and/or precipitation preferences, while their frequency appears higher among older events. This suggests that adaptation to different substrates evolved early in New Caledonian palms, which is different from what has been found for *Oxera* (Barrabé *et al.*, 2019) and *Diospyros* (Paun *et al.*, 2016). Although the ancestral state inferences tend to suggest that most transitions are from ultramafic to non-ultramafic substrates (Fig. 3), this remains to be confirmed by studies including more data for non-New Caledonian palms. The three speciation events apparently not associated with any ecological or geographical transition that were found in *Basselinia* (in comparison to only one among the other New Caledonian palms) suggest that additional factors might have played a more prominent role in the diversification of this comparatively species-rich genus (14 species). Such factors might include flowering time or more specific climatic differences (e.g. precipitation during the dry season). Our results are in line with the findings of Barrabé *et al.* (2019), who showed that a combination of different factors could be behind the speciation processes of plants in New Caledonia, and with the suggestion of Pintaud *et al.* (2001) that palm speciation in New Caledonia resulted from multiple factors. The fact that allopatric and parapatric speciation and ecological divergence could contribute to explaining the current diversity of palms in New Caledonia is consistent with the findings of Paun *et al.* (2016) for *Diospyros* (Ebenaceae). In contrast, it appears that substrate has not played a major role in the most recent diversification of New Caledonian palms, despite facilitating speciation in other New Caledonian plant groups, such as the nickel hyperaccumulating species of *Geissois* (Cunoniaceae; Pillon *et al.*, 2014).

## Conclusion

Our study provides the first well-resolved, comprehensive spatio-temporal framework for New Caledonian palms and their relatives across Southeast Asia and the Pacific. Our estimate of phylogenetic relationships among New Caledonian palms species, the most robust yet available, provides important support for the prevailing classification while pinpointing issues, such as the resurrection of *Campecarpus* and *Veillonia* and the need for further study of Rhopalostylidinae relative to Basseliniinae. Our biogeographical analyses reveal the prominent role played by New Guinea lineages in shaping New Caledonian palm diversity (via Australia in the case of Archontophoenicinae) and highlight New Caledonia as a source of palm diversity for the neighbouring regions, especially the Pacific Islands. We show that the *in situ* speciation that led to high endemism in New Caledonian palms was likely driven by multiple factors, with substrate having played a role only during the early evolutionary history of the group, whereas ecological and range transitions are more likely to have driven recent speciations, such as in *Basselinia*.

These results provide a model and baseline for future studies of diversification and its ecological drivers in New Caledonian plants. In particular, investigations of genetic and ecological differentiation between recently diverged species (such as in *Basselinia*) might provide insights into the mechanisms underpinning radiations in this hotspot of endemicity. Our results challenge the idea that New Caledonia is a stable refuge for old lineages, underlining instead the dynamism of the island and its contribution to the formation of plant diversity in the Pacific region.

## SUPPLEMENTARY DATA

Supplementary data are available at *Annals of Botany* online and consist of the following.

Figure S1: phylogenetic relationships among New Caledonian arecoid palms and closely related genera obtained by multispecies coalescent summary analysis of 151 gene regions with gene tree branch weighing (Weighted ASTRAL). Numbers represent the local posterior probability of each clade. Red numbers indicate branching patterns that differed from the species tree obtained without branch weighing presented in Fig. 1. Figure S2: divergence times and ancestral ranges of New Caledonian arecoids and closely related genera. Ancestral ranges were inferred under a DEC+J model including an area distance matrix. Pie charts represent the probability of each state, with areas colour coded as shown on the top left map. Tip squares indicate the current distribution of each taxon, with areas colour coded following the top left map and letter coded as described in the Materials and Methods and the Supplementary Data (Table S1). New Caledonian lineages are highlighted in grey, and the current taxonomic classification is specified on the right. Genera without a subtribe name are unplaced according to Baker and Dransfield (2016). Grey vertical bars indicate dry periods in the Southwest Pacific following Chamley (1986). Incongruences with Fig. 2 are indicated by asterisks. The letters B, C and D indicate the clades shown in panels B, C and D of Fig. 1. Figure S3: divergence times and their 95 % highest posterior density intervals. Figure S4: distribution range of New Caledonian arecoid palms. Red polygons indicate the suggested Pleistocene refugia according to Pintaud *et al.* (2001). Table S1: samples included in this study, with information on vouchers, geographical distribution, number of reads, gene recovery and Illumina data GenBank accession numbers. Table S2: genes selected for the molecular dating, with their nucleotide substitution model and length. Table S3: annual precipitation and elevation for each occurrence point. Table S4: biogeographical models, with their AICc values.

mcae043_suppl_Supplementary_Materials
